# Supplementary material for: MetaRibo-Seq measures translation in microbiomes
Source: Nat Commun. 2020 Jun 29;11:3268. doi: 10.1038/s41467-020-17081-z (PMC7324362; doi:10.1038/s41467-020-17081-z)
Supplement: Supplementary file 10 — Supplementary Data 7 [file 41467_2020_17081_MOESM10_ESM.zip › File2/Confidence_VeryHigh_Taxonomy/351966_out.krona.html]

Javascript must be enabled to view this page.

members
magnitude
magnitudeUnassigned
count
unassigned
taxon
rank

351966\_out

9

2
9
superkingdom

1239
9
phylum

186801
9
class

9
186802
order

family
9
31979


SRS054956\_contig\_number\_contig-100\_13327.50969
genus
1
9
1485

2293029

SRS054590\_contig\_number\_1200SRS057717\_contig\_number\_contig-100\_8098.34689SRS147139\_contig\_number\_39675
3
species

species
2293024
2

SRS077552\_contig\_number\_23619SRS147919\_contig\_number\_contig-100\_2858.88849

2293054

SRS143070\_contig\_number\_contig-100\_31332.31332
1
species

1262832

SRS147766\_contig\_number\_19455
1
species

1

SRS144714\_contig\_number\_17731
2293025
species
